# The long path to pregnancy: early experience with dual anonymous gamete donation in a European in vitro fertilisation referral centre

**DOI:** 10.1186/1742-4755-7-20

**Published:** 2010-08-11

**Authors:** Eric Scott Sills, Lyubov O Mykhaylyshyn, Ulyana S Dorofeyeva, David J Walsh, Umme Salma, Ahmed B Omar, Graham D Coull, Ileana A David, Kathy M Brickell, Olga M Tsar, Anthony PH Walsh

**Affiliations:** 1 Division of Reproductive Endocrinology, The Sims Institute/Department of Obstetrics & Gynaecology, School of Medicine, Royal College of Surgeons in Ireland; Dublin, Ireland; 2 IVF Unit, Intersono Clinic Ltd; Lviv, Ukraine; 3 Sims IVF, Rosemount Hall, Dundrum Road, Dundrum, Dublin 14 Ireland

## Abstract

**Background:**

This investigation describes features of patients undergoing in vitro fertilisation (IVF) and embryo transfer (ET) where both gametes were obtained from anonymous donors.

**Methods:**

Gamete unsuitability or loss was confirmed in both members of seven otherwise healthy couples presenting for reproductive endocrinology consultation over a 12-month interval in Ireland. IVF was undertaken with fresh oocytes provided by anonymous donors in Ukraine; frozen sperm (anonymous donor) was obtained from a licensed tissue establishment. For recipients, saline-enhanced sonography was used to assess intrauterine contour with endometrial preparation via transdermal estrogen.

**Results:**

Among commissioning couples, mean±SD female and male age was 41.9 ± 3.7 and 44.6 ± 3.5 yrs, respectively. During this period, female age for non dual anonymous gamete donation IVF patients was 37.9 ± 3 yrs (*p *< 0.001). Infertility duration was ≥3 yrs for couples enrolling in dual gamete donation, and each had ≥2 prior failed fertility treatments using native oocytes. All seven recipient couples proceeded to embryo transfer, although one patient had two transfers. Clinical pregnancy was achieved for 5/7 (71.4%) patients. Non-transferred cryopreserved embryos were available for all seven couples.

**Conclusions:**

Mean age of females undergoing dual anonymous donor gamete donation with IVF is significantly higher than the background IVF patient population. Even when neither partner is able to contribute any gametes for IVF, the clinical pregnancy rate per transfer can be satisfactory if both anonymous egg and sperm donation are used concurrently. Our report emphasises the role of pre-treatment counselling in dual anonymous gamete donation, and presents a coordinated screening and treatment approach in IVF where this option may be contemplated.

## Background

For some patients, parenthood hinges on the successful completion of a medical odyssey comprising a fretful maze of tests and procedures. Among the advanced reproduction techniques currently available, one of the more complicated is anonymous donor oocyte IVF. The complexity and cost of this treatment relates to the interlock of screening and clinical management of the oocyte donor, the embryo recipient, and the sperm source (usually, the recipient's partner). Although anonymous donor oocyte IVF generally requires eggs to be collected fresh, since cryopreserved spermatozoa have long been available from tissue establishments ("sperm banks") this has made anonymous acquisition of male gametes comparatively simple. But while the frequency of IVF incorporating either donor oocyte [1] or donor sperm has trended upward recently, dual anonymous gamete donation has rarely (if ever) been previously reported. With a view to characterise this type of fertility treatment better, our investigation sought to provide a clinical summary of IVF couples who undergo dual anonymous gamete donation.

## Methods

Medical records were reviewed for all in vitro fertilisation (IVF) cases at Sims International Fertility Clinic (Sims IVF/Dublin, Ireland) to identify cases where both oocyte and sperm from anonymous gamete donors was used during treatment. We studied de-identified IVF records of all couples who completed embryo transfer during 2009. Given the emerging regulatory environment for assisted fertility services in Ireland, cycle commencement and monitoring occurred in Dublin, while embryo transfer was performed at our affiliate clinic site in Ukraine (Intersono, Lviv).

IVF patients were considered for dual anonymous gamete donation if prior donor oocyte IVF treatment using partner sperm had failed. However, in cases where prior ovarian response to gonadotropin ovulation induction was poor and where the male partner evaluation also identified severe abnormality with semen parameters and/or sperm DNA fragmentation (and no donor gametes were used), couples could request to move directly to dual anonymous gamete donation without first having to complete one cycle of anonymous donor sperm or egg IVF. Anonymous oocyte donors underwent comprehensive medical and psychological evaluation in Ukraine, as described previously [2]. Recipients had their initial reproductive endocrinology consultation and all pre-embryo transfer monitoring in Ireland. Anonymous oocyte donor counselling was provided by an accredited psychologist before beginning gonadotropins in Lviv; recipients and their partners completed parallel counselling in Dublin. All recipient couples participating in dual anonymous gamete donation had already matriculated into Ireland's national adoption counselling protocol. These patients were in various stages of assessment as adopters, involving a comprehensive extramural evaluation designed to prepare them for parenthood via adoption. The process to adopt a child had therefore triggered a recommendation letter from consultants in support of the couple's adoption application, and redacted summaries of those counselling narratives were evaluated for this study.

After undergoing further counselling specifically on dual anonymous gamete donation, couples were offered this treatment as an alternative to adoption. They were apprised of the procedure's complexity and the international nature of the treatment was acknowledged. Each recipient selected an anonymous oocyte donor via secure internet portal with an electronic lock-out mechanism to prohibit multiple recipients from accessing the aggregate donor pool at the same time. Following registration of each provisional donor-recipient match, the corresponding anonymous oocyte donor entry was deleted from the donor library [2], thus creating a 1:1 ratio for each recipient and their anonymous oocyte donor. Anonymous sperm samples were individually ordered from a pre-screened cryobank inventory provided by a licensed tissue establishment. No two recipients requested anonymous sperm from the same anonymous donor.

After match verification and institutional receipt of anonymous (frozen) donor sperm, the anonymous oocyte donor commenced controlled ovarian hyperstimulation. Programmed transvaginal ultrasound-guided oocyte collection followed 36 h after s.c. hCG administration. Anonymous donor sperm was used to fertilise all freshly retrieved eggs obtained from the anonymous oocyte donor; intracytoplasmic sperm injection (ICSI) was performed in all cases due to high variation in post-thaw sperm motility. The recipient underwent endometrial preparation by transdermal estrogen. Fresh embryo transfer occurred at the blastocyst stage under abdominal ultrasound guidance, and any non-transferred embryos were cryopreserved [3]. Supplementary luteal phase support was provided for all recipients, who returned for hCG testing 12-14 d after embryo transfer. Progesterone was discontinued for non-pregnant patients. After oocyte retrieval, donor health and overall satisfaction with the anonymous donation process was elicited by nursing and medical staff. Clinical pregnancies among recipients were recorded for analysis.

For all dual donation couples, recipient and partner age were tabulated as was age of the anonymous oocyte donor (the ages of anonymous sperm donors were not ascertained). The following laboratory parameters were also evaluated: number of oocytes collected from anonymous donor, number of 2*pn *embryos generated after fertilisation, number of embryos transferred, and number of non-transferred cryopreserved embryos. Additionally, infertility duration and history of prior fertility treatments were recorded for all recipients.

## Results

A total of 1,003 non-donor embryo transfer cycles were completed at the monitoring site during the 12-month study interval. During this period, mean±SD age of all patients initiating IVF treatment at the monitoring site was 37.9 ± 3 yrs. Couples embarking on IVF with dual anonymous gamete donation (*n *= 7), had mean±SD female age of 41.9 ± 3.7 yrs. Male age was 44.6 ± 3.5 yrs in this group (dual anonymous gamete donation female recipient age vs. general IVF female patient age, *p *< 0.001 by Student's *t*-test).

For all females who enrolled in dual anonymous gamete donation, infertility duration was at least 3 yrs. All these couples had at least two prior failed fertility treatments using native oocytes, and five of seven couples had already completed one unsuccessful cycle of anonymous donor oocyte IVF (using partner sperm). The other two couples (who had not done anonymous donor oocyte IVF previously) had experienced total fertilisation failure of native oocytes when partner sperm was used for IVF+ICSI. In these two cases, both oocyte quantity and quality were profoundly impaired and the partner's sperm DNA fragmentation exceeded 40%. Although the problem of bilateral gamete impairment could have been remedied in a stepwise fashion by the incremental introduction of either anonymous donor sperm or oocytes for IVF, this therapeutic option was rejected by these two couples.

**Figure 1:**
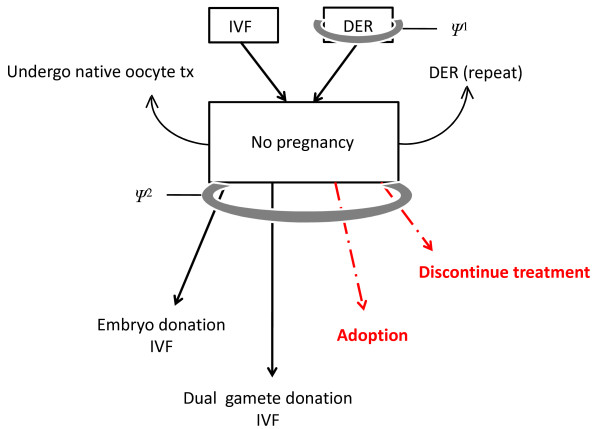
**The IVF experience: pathways after failure**. Schematic of options for study patients failing to achieve pregnancy after in vitro fertilisation (IVF), including those with prior experience with anonymous donor oocyte (DER) treatments. Non-medical pathways are shown in red. Gray bands indicate mandatory psychological counselling interventions at various treatment stages (Ψ^1 ^and Ψ^2^).

All recipient couples were familiar with traditional adoption in Ireland; there was no uniform reason why couples decided not to pursue adoption. Uncertainty about (or disagreement with) intercountry adoption policy in Ireland was expressed by 6 of 7 couples who embarked on IVF with dual gamete donation. Additional counselling focusing on dual anonymous gamete donation found the seven couples to be good candidates for this treatment, and since adoption in Ireland is typically an international transaction anyway, the Ukrainian component was not considered objectionable. Figure 1 summarises options considered by fertility patients in this sample.

For anonymous oocyte donors, mean±SD age was 27.1 ± 3 yrs, and all were Caucasian non-smokers with at least one child from an unassisted pregnancy. Recipients in Ireland were matched to their first-choice anonymous oocyte donor in Ukraine in each case, although one patient completed two dual-donor IVF cycles using a different anonymous oocyte donor for her second attempt. Within six weeks of anonymous donor accession, screening was completed for all anonymous oocyte donors in accordance with the European Union Tissues & Cells Directive [2004/23/EC]; a karyotype was obtained on each donor in addition to statutory requirements [2]. No aneuploidy was identified in any donor from this series. Anonymous donor sperm was commercially obtained in standard cryovials from a licensed tissue establishment, and arrived at the Ukrainian clinical site within four weeks of order placement.

From anonymous oocyte donors, an average of 21 oocytes was obtained (range = 10-24). After fertilisation with anonymous donor sperm, 17.6 ± 4.5 advanced to the 2*pn *stage. Mean number ±SD of embryos transferred in this series was 2.1 ± 0.3, and commissioning couples had an average of 7.6 non-transferred embryos cryopreserved for storage (range = 1-17). Embryo transfer was completed in all seven cases, but one couple underwent two transfers. Clinical pregnancy was achieved for five (5/7 = 71.4%) patients. Transvaginal ultrasound at 7-10 weeks' gestation confirmed an intrauterine pregnancy for five patients, and two of these had twins (40% multiple gestation rate/transfer, see Table 1).

**Table 1:** Summary of clinical and laboratory characteristics observed among couples undergoing IVF with dual anonymous gamete donation

Commissioning couple		Oocyte donor age (yrs)	Oocytes retrieved (*n*)	#ET	#embryos frozen	outcome^1^
					
Female age (yrs)	Male age (yrs)					
42	50	32	13	2	0	0
		27	25	2	17	0
45*	45	28	18	2	9	2
44*	45	28	21	3	4	1
43	43	28	24	2	10	0
39	39	23	21	2	12	1
45	43	24	22	2	6	1
35	47	24	22	2	6	2

41.9 ± 3.7	44.6 ± 3.5	26.4 ± 2.9	21 ± 3.6	2.1 ± 0.3	7.6 ± 5.1	

*Notes*: Data reported as mean ± SD. ET = embryo transfer. The first case was matched to two different anonymous oocyte donors, but has yet to attain pregnancy (17 cryopreserved embryos remain in storage for this couple)

(*) no history of anonymous donor gamete therapy before enrolling into dual anonymous donor gamete IVF treatment

^1 ^Classification system for reproductive outcome: 0 = no pregnancy; 1 = singleton intrauterine pregnancy; 2 = twin pregnancy. Overall clinical pregnancy rate/ET = 62.5%

## Discussion

Donor gamete IVF typically joins the path to pregnancy as a late entry. As a fertility treatment generally reserved for the most refractory cases, anonymous donor oocyte IVF is usually preceded by multiple failed treatment cycles. For such patients, substantial emotional stress may coalesce around donor gamete IVF because of feelings of reproductive inadequacy or disappointment. Indeed, some fertility patients never accept the donor gamete alternative and steadfastly resist any reproductive option involving gamete donation [4]. These psychological factors should not be underestimated; they often figure prominently in the decision to disengage from fertility treatments [5,6]. Remarkably, the stress release achieved by stopping all fertility treatment can be followed by an unassisted conception, even at advanced age [7].

While previous investigations have focused on either anonymous donor oocyte or sperm with IVF, this work is the first to collect data on an IVF patient series where both gametes derived from anonymous donors. Irrespective of the clinical indication(s) leading to use of donor gametes with IVF, this treatment modality brings unique and sensitive issues not encountered with other assisted fertility pathways. For example, a successful anonymous donor oocyte IVF cycle may introduce family concerns regarding a "separated biological and social parenthood" [8], and feelings of fathers who use anonymous donor sperm to establish their family have been similarly depicted [9]. Considerable research has also focused on how anonymous oocyte [10] and sperm [11] donors regard the total donation experience. The anonymous donor has been variously portrayed as the shadowy and ambiguous figure of 'another man', the intelligent medical student, or the donor as 'family man', with children of his own who wants to help infertile men father children [9]. These diverse sentiments underscore the essential requirement of psychological counselling for all parties before commencing any fertility treatment incorporating donated gametes [12]. Given the psychological issues that can accompany IVF when a single gamete donor is used, it is plausible that such factors are compounded when both gametes used in IVF are anonymously donated.

It should be noted that IVF with dual anonymous gamete donation shares some features with embryo donation, in that neither provides a genetic linkage between parents and offspring. Key differences must be acknowledged between the two approaches, however. For example, in IVF with dual anonymous gamete donation, the commissioning couple initiates the entire treatment sequence *de novo*. In contrast, donor embryo IVF is conditional on the availability of surplus embryos derived from someone else's prior IVF treatment. Studies on family dynamics after donor embryo IVF have suggested higher emotional overinvolvement and defensive responding are more often observed along with greater secrecy about the child's origins [13]. Moreover, a gestational connection between mother and child exists when either oocyte donation or embryo donation IVF succeeds, allowing a mother to feel that the child is "hers" and that she is a "normal" mother who conceived "naturally" [14]. Notably, anonymous oocyte donation and embryo donation enable patients in both IVF groups to obscure their lack of genetic relationship to offspring, friends, or family [15]. Some of these characteristics might be generalisable to families where IVF with dual gamete donation was used successfully, although this issue awaits further study.

Perhaps not surprisingly, our research on IVF with dual anonymous gamete donation revealed that each couple undergoing this treatment develops their own narrative describing how this key decision is made. A theme of "engagement with technology" as a means to attain parenthood was commonly identified in recipient counselling sessions here. While the notion of dual gamete donation IVF was never portrayed as an ideal route to pregnancy, the alternative of childlessness was clearly regarded as unacceptable. Moreover, all seven recipient couples were conversant with traditional adoption and none objected to having a family in this way (some actually preferred traditional adoption over medical interventions like IVF). But in Ireland, a government-regulated state adoption scheme does not favour continuation of fertility treatments in parallel to the adoption process. Curiously, while this agency "encourages" prospective adopters "to complete fertility investigations prior to pursuing adoption" [16], in practice all patients in our study group had been told to stop fertility treatments while on the adoption wait-list, or risk being disqualified for adoption. As Irish legislation to address assisted reproductive treatments is contemplated, policies of making traditional adoption contingent on an applicant's discontinuation of all clinical assistance to achieve a pregnancy should be re-examined.

A novel feature of this dual anonymous donor gamete IVF programme is that neither gamete donor was of Irish origin. As previously reported, the paucity of domestic anonymous gamete donors in Ireland together with the regulatory vacuum with respect to all assisted fertility treatments frustrates the provision of this service here [2]. For these couples, the role of the clinical site in Ireland was limited to cycle monitoring while (anonymous donor) oocyte retrieval and embryo transfer were performed in Ukraine. This circumstance may change when Ireland implements comprehensive legislation governing assisted reproduction that addresses anonymous gamete donation. Considerable public debate has already transpired in other jurisdictions about the wishes of donor-conceived people, as this group has gained a voice regarding their experience of not being able to access basic information about their genealogical heritage [17]. The rights and needs of donor-conceived people regarding their access to identifying information about their genetic parents [18] have special relevance when neither "social" parent has a genetic connection to their children, as in IVF with dual anonymous gamete donation. The treatment we describe here brings forward themes similar to those identified with donor oocyte IVF, regarding parenthood as well as the cognitive and socioemotional development of the resulting offspring [19]. Longitudinal studies of children resulting from anonymous donor gamete IVF have particularly focused on disclosure (openness) vs. non-disclosure (secrecy) [20]. Earlier research has suggested that many infertile couples who have benefited from assisted fertility treatments using donated gametes have no intention to disclose this fact to their offspring [21,22]. Indeed, our patients variably expressed a "protective worry" that if disclosure were to be made compulsory, the reaction or attitude of the donor/s at some point in the future would be unpredictable, and might not be positive for a previously unknown child conceived from their gametes. Interestingly, this tendency to maintain confidentiality has been noted even among fertility patients in countries where legislation mandates disclosure of donor identity when the offspring reach adulthood [23]. These investigations underscore the role of careful pre-treatment counselling which, among other things, helps anticipate parenting strategies for would-be parents after particular fertility interventions.

## Conclusions

Should anonymous gamete donation be restricted by statute in Ireland, then Irish patients who specifically seek this treatment may travel elsewhere for this reproductive option. Evidence exists supporting such a consequence in the E.U., as in Sweden sperm donors are required by law to be identifiable, while anonymous sperm donation is permitted in Denmark. The shortage of Swedish sperm donors has contributed to cross-border "reproductive tourism" between Sweden and Denmark [24]. How regulation of clinical treatments incorporating anonymous donor gametes in Ireland will develop is uncertain, but the current research offers early insights regarding patients most directly impacted by such legislation.

## Competing interests

The authors declare that they have no competing interests.

## Authors' contributions

ESS was principal investigator and lead author; LOM and USD were chief clinicians at the Ukrainian site; DJW was senior reproductive endocrinologist; US, ABO and GDC were programme associates in Dublin; IAD and KMB were nurse coordinators in Dublin; OMT was donor welfare coordinator; APHW was chief of service and supervised overall research design. All authors read and approved the final manuscript.
